# A Web-Based Form With Interactive Charts Used to Collect and Analyze Data on Home Births in Italy

**DOI:** 10.2196/10335

**Published:** 2019-03-22

**Authors:** Michele Zanetti, Rita Campi, Paola Olivieri, Marta Campiotti, Alice Faggianelli, Maurizio Bonati

**Affiliations:** 1 Laboratory for Mother and Child Health Department of Public Health Istituto di Ricerche Farmacologiche Mario Negri Milan Italy; 2 Associazione Nazionale Culturale Ostetriche Parto a Domicilio e Casa Maternità Varese Italy

**Keywords:** Web-based form, home birth, interactive charts, internet, survey methods

## Abstract

**Background:**

The use of Web-based forms and data analysis can improve the collection and visualization of data in clinical research. In Italy, no register exists that collects clinical data concerning home births.

**Objective:**

The purpose of this study was (1) to develop a Web portal to collect, through a Web-based form, data on home births in Italy and (2) to provide those interested with a graphic visualization of the analyses and data collected.

**Methods:**

Following the World Health Organization’s guidelines, and adding questions based on scientific evidence, the case report form (CRF) on the online form was drafted by midwives of the National Association of Out-of-Hospital Birth Midwives. During an initial phase, a group of midwives (n=10) tested the CRF, leading to improvements and adding the necessary questions to achieve a CRF that would allow a more complete collection of data. After the test phase, the entire group of midwives (n=166) registered themselves on the system and began filling out birth questionnaires. In a subsequent phase, the administrators of the portal were able to view the completed forms in a graphic format through the use of interactive maps and graphs.

**Results:**

From 2014 to 2016, 58 midwives included 599 birth questionnaires via the Web portal; of these, 443 were home-based, 76% (321/424) of which were performed at home and 24% (103/424) at a midwifery unit. Most of the births assisted (79%, 335/424) were in northern Italy, and the average ages of the mother and father were 33.6 (SD 4.7) years and 37.0 (SD 5.6) years, respectively.

**Conclusions:**

We developed an innovative Web-based form that allows, for the first time in Italy, the collection of data on home births and births in the midwifery unit. Furthermore, the data collected are viewable online by the midwives through interactive maps and graphs that allow them to have a general and continuously updated view of the situation of out-of-hospital births performed by the National Association of Out-of-Hospital Birth Midwives. The future goal is to be able to expand this data collection to all out-of-hospital births throughout the national territory. With an increase in the number of enrolled midwives, it would be possible to use the portal as a Web-based form and also as a portal for sharing resources that would help midwives in their clinical practice.

## Introduction

From the mid-20th century, giving birth moved from the home into the hospital in most well-resourced countries. This change was related to the decreased rates of perinatal, neonatal, and maternal mortality observed in the same period. In-hospital birthing was one of the possible explanatory causes, perhaps the most important, as even living conditions, in general, began to change at that time. The rapid improvement of medical technologies and the availability of more knowledge and resources led to the over-medicalization of procedures once considered natural and physiological events, such as childbirth, which needs few obstetrical interventions when of low risk [1]. Thus, although birthing in a hospital is the cultural norm, in recent decades in a few countries, the number of out-of-hospital births is increased [2-4].

Rates of planned out-of-hospital births (ie, births intended to occur at home or at a freestanding birth unit) are low in high-income countries, although they vary widely. The highest rate is in the Netherlands; in the past, almost 30% of all babies born here were born at home, and home birth had always been an integrated part of the maternity system. Women in the Netherlands also currently have the option of giving birth in a birth center (a home-like setting); 11.4% of births occur in this setting and 16.3% at home [5,6]. In Wales, England, Scotland, Iceland, and Switzerland, out-of-hospital birth rates are 1% to 3%, whereas in other European countries rates are lower at 1% [5]. In the United States, although it recently increased, the rate is around 1.5% [7].

The Certificate of Delivery Assistance (CeDAP) is the nationwide mandatory questionnaire, completed by the midwife or physician attending the delivery, which collects information about the parents, the pregnancy, and the newborn baby. The CeDAP constitutes the richest source of health, epidemiological, and sociodemographic information concerning the birth event. This certificate is not always complete for out-of-hospital births and therefore does not contain all the information necessary to better assess the quality of births performed at home or in a midwifery unit.

**Figure 1:**
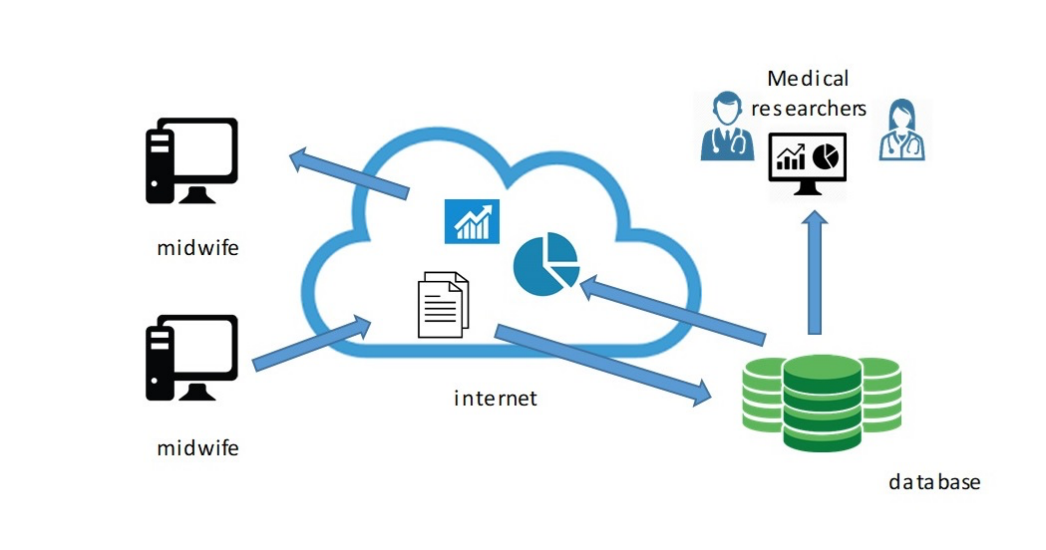
Data flow overview.

To obtain more information on and to evaluate out-of-hospital births, it was necessary to provide the birth assistance certificate with additional questions; for this reason, a few years ago we began to collect paper data sheets and PDF forms. However, problems were found during collection. In fact, to be processed, the data collected had to be sent by mail to the processing site, which involved a waste of resources. In the last few years, the paper data collection questionnaires have been joined by increasingly advanced digital data collection systems: Web pages allow users to enter data collected in their studies and share it immediately with other users or simply analyze it for their own purposes (Figure 1).

The continuous development of information technology has allowed us to improve the computerized data collection questionnaires further, making them increasingly user-friendly for those entering the data collected during clinical practice.

With this information and with the help of the National Association of Out-of-Hospital Birth Midwives (called “the association” from here on), we decided to develop a portal [8] that would permit the collection of data on home births in Italy by the midwives who belong to the association.

## Methods

### Recruitment

The association is a national network of qualified midwives providing care and support to pregnant women and their babies before, during, and after childbirth. The association, founded in 1981, covers almost all midwives who attend out-of-hospital births. All qualified midwives who adhere to the association assist women who meet the eligibility criteria for out-of-hospital birth defined by national and international guidelines [9]. For the data acquisition, all 166 midwives who are part of the association participated. The midwives work privately, outside the national health system. They assist births at home or in private units run by midwives (a midwifery unit).

### Portal Development

The portal was developed using ASP.NET WebForms, an open source Web framework for building modern Web apps and services with .Net. For data management, it used Microsoft SQL SERVER 2008 R2, a relational database management system with which all information was saved, loaded, and managed. In the database, the data from the forms were saved in JSON (JavaScript Object Notation) format.

### Form Development

**Figure 2:**
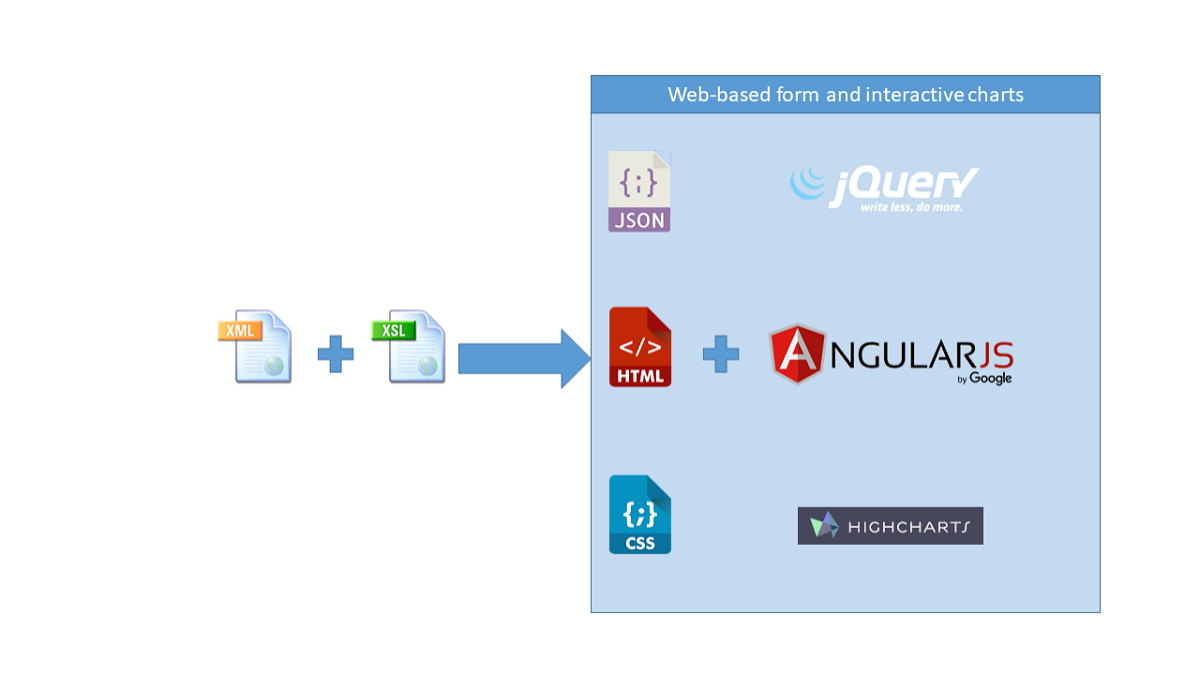
The case report form system developed.

Case report form (CRF) variables were defined in an eXtensible Markup Language (XML), then automatically transformed into an HTML page using XSLT transformation; the HTML page contains tags and attributes managed by AngularJS, a framework JavaScript client-side compatible with both desktop and mobile browsers [10]. All data services (loading and saving) were handled via ANGULARJS and JQUERY as seen in Figure 2.

The XML, which contains the CRF variables, can be changed at any time and according to need (eg, additional CRF versions, new variables, bug detection) without having to rewrite other files or change the database structure. All the architecture described is downloadable from GitLab [11].

### Data Collection

**Figure 3:**
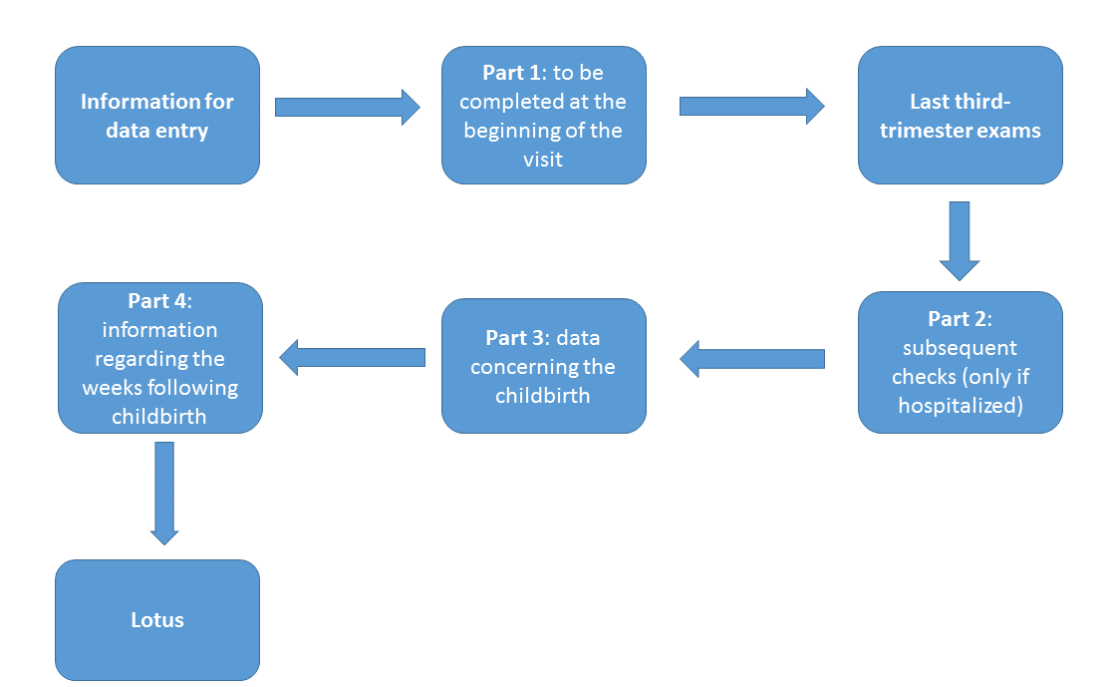
Case report form block diagram.

Following the World Health Organization’s guidelines, and adding questions based on scientific evidence, the CRF was created by the midwives of the association. The CRF records every moment of pregnancy from the first visit to the end of each birth, including the weeks immediately following the birth (Figure 3). The main parts of the online questionnaires are described subsequently.

- *Information person completing form*: information regarding the midwife filling out the form. It is not necessarily the same person who followed the birth, but the general information is required.
- *First part*: to be completed at the beginning of the visit. Some information on the mother is collected (eg, age, diet, blood pressure).
- *Last third-trimester exams*: a report of the tests performed (eg, blood tests, fetal presentation).
- *Second part*: subsequent checks (only in the event of hospitalization). This part collects data on whether the exclusion from home care took place before labor. If the answer is affirmative, a series of questions must be filled out regarding the outcome of the childbirth and the motivations that led to exclusion.
- *Third part*: essential data concerning the childbirth (eg, place, date, time).
- *Fourth part*: information regarding the weeks following childbirth, type of breastfeeding, and whether the child has had any problems.
- *Lotus*: asks if lotus birth has been carried out. Lotus birth is the practice of leaving the umbilical cord uncut so that the baby remains attached to the placenta until the cord naturally separates at the navel as a cut cord does 3 to 10 days after birth. If a lotus birth was carried out, information on type of lotus and related information is collected, including the parent’s experience.

### Interactive Charts

The compilation of the questionnaires by midwives has made it possible to obtain a source of useful information not only for the group of clinicians involved in the processing of data but also for the midwives of the association. To facilitate the reading of the data, we created an area of the portal dedicated to the graphic visualization of the collected data. This area was created using Highcharts, a scalable vector graphics-based, multiplatform charting library, which makes it easy to add interactive charts [12].

**Figure 4:**
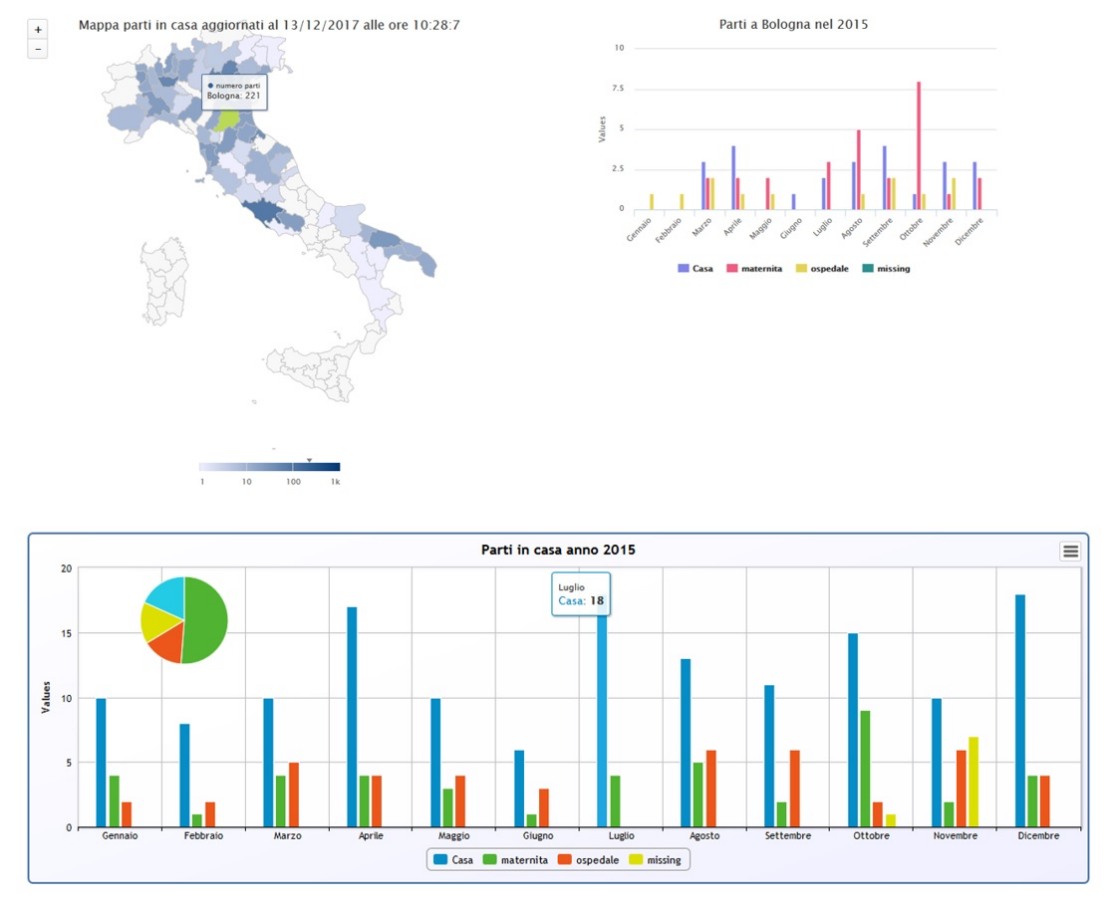
Interactive charts of births. The map of Italy is divided into provinces, colored differently based on the number of questionnaires. Selecting a single province provides information about the births in that area by month in a bar graph (top right). The bottom graph shows births across Italy by month and place of birth.

On one page, for example, a map of Italy is divided into provinces, which are colored differently based on the number of questionnaires. By selecting a single province, information about the births in that area can be visualized by month and by place of birth as seen in Figure 4 the bar graph in the top right. The bottom graph shows births across Italy by month and place of birth.

### Data Processing

#### Statistical Methods

The results presented compare women who gave birth at home to those who gave birth in a freestanding midwifery unit.

Categorical variables were summarized using proportions and associations tested using chi square or Fisher exact tests, where applicable. Continuous variables were summarized using means and standard deviations for normally distributed data, whereas skewed data were summarized using medians. A two-tailed independent *t* test was used to test differences of means for normally distributed continuous variables; the Mann-Whitney *U* test was used for skewed continuous variables.

To identify risk factors, we computed relative risks (RRs) using a multivariate log-binomial regression model, considering the significance of the confidence intervals. Statistical significance was evaluated using 95% confidence intervals and a two-tailed *P* value of <.05.

All data management and analyses were performed using SAS software.

## Results

Data were collected on 424 Italian women who delivered out-of-hospital, 321 (75.7%) of whom delivered at home and 103 (24.3%) at a freestanding midwifery unit. The majority of recruited deliveries (79.0%, 335/424) took place in northern Italy. The mean ages of the mothers and fathers were 33.6 (SD 4.7) years and mean 37.0 (SD 5.6) years, respectively (Table 1). The mothers had a higher level of education than the fathers, and the fathers worked more often (99%, 413/424) than mothers (78%, 331/424). The distributions of mothers for parity, age at first delivery, and educational level were different between mothers who delivered at home and those who delivered in a freestanding midwifery unit. In the multivariate regression model, being a primipara and having the first child at age 35 years or older was associated with a slightly higher risk of delivering in a freestanding midwifery unit compared to at home (age <35 years: RR 1.9, 95% CI 1.1-3.2; age ≥35 years: RR 2.3, 95% CI 1.1-4.8).

Of the 247 multiparas, the majority (63.2%, 156/247) had previously given birth in a hospital, and most had a normal vaginal birth (91.9%, 227/247). The majority of multiparas who delivered at home (76.1%, 188/247) had previously given birth in a hospital, whereas about half of the multiparas who delivered in a freestanding midwifery unit (51.4%, 127/247) had previously given birth at home.

A quarter of the mothers reported desire for intimacy as a reason for delivering out-of-hospital. Positive previous experience at home and a desire for a new experience were the other frequent reasons for justifying the choice of delivering at home, whereas naturalness and a trusting relationship with midwives were the main reasons for choosing to deliver in a freestanding midwifery unit.

**Table 1:** Characteristics of women having planned births at home or in a freestanding midwifery unit, and of their partners.

Characteristic			Delivery location			Relative risk (95% CI)	*F* (*df*1,*df*2)	*P* value
			At home (n=321)	In a freestanding midwifery unit (n=103)	Overall (N=424)			
**Maternal characteristics**								
	Age (years), mean (SD)		33.5 (4.6)	33.9 (4.9)	33.6 (4.7)		1.13 (320,102)	.41
	**Age group (years), n (%)**							
		18-24	9 (2.8)	3 (2.9)	12 (2.8)	0.98 (0.70-1.37)		
		25-34	171 (53.3)	52 (50.5)	223 (52.6)	Reference		
		≥35	141 (43.9)	48 (46.6)	189 (46.6)	0.97 (0.87-1.09)		
	**Residential area, n (%)**							
		Large city	117 (42.1)	41 (50.0)	158 (43.9)	Reference		
		Medium-size city	111 (39.9)	21 (25.6)	132 (36.7)	1.14 (1.01-1.28)		
		Small town	50 (18.0)	20 (24.4)	70 (19.4)	0.96 (0.81-1.15)		
		Missing	43	21	64			
	**Marital status, n (%)**							
		Married and/or cohabiting	229 (71.3)	74 (71.8)	303 (71.4)	Reference		
		Other	92 (28.8)	29 (28.2)	121 (28.6)	1.25 (1.10-1.42)		
	**Number of children, n (%)**							
		First	100 (32.9)	51 (54.3)	151 (37.9)	Reference		
		Second or more	204 (67.1)	43 (45.7)	247 (62.1)	1.25 (1.10-1.42)		
		Missing	17	9	26			
	**First delivery >35 years, n (%)**							
		Yes	22 (6.9)	20 (19.4)	42 (9.9)	0.67 (0.50-0.90)		
		No	299 (93.1)	83 (80.6)	382 (90.1)	Reference		
	**Level of education, n (%)**							
		Primary	5 (1.6)	3 (2.9)	8 (1.9)	Reference		
		Secondary	102 (31.9)	29 (28.2)	131 (31.0)	1.25 (0.72-2.15)		
		Postsecondary	214 (66.6)	71 (68.6)	285 (67.1)	1.20 (0.70-2.06)		
	**Occupational status before index birth, n (%)**							
		Working	246 (76.6)	85 (82.4)	331 (78.0)	Reference		
		Not working	75 (23.4)	18 (17.6)	93 (22.0)	1.08 (0.96-1.22)		
	**Annual income (€), n (%)**							
		<20,000	41 (15.1)	10 (10.5)	51 (13.9)	Reference		
		20,000-29,000	109 (40.1)	33 (34.7)	142 (38.7)	0.95 (0.81-1.12)		
		≥30,000	122 (44.9)	52 (54.7)	174 (47.4)	0.57 (0.74-1.03)		
		Missing	49	8	57			
	**Diet, n (%)**							
		Omnivorous	238 (74.1)	80 (77.7)	318 (75.0)	Reference		
		Other	83 (25.9)	23 (22.3)	106 (25.0)	1.05 (0.93-1.18)		
	**Smoker, n (%)**							
		Yes	10 (3.1)	7 (6.8)	17 (4.0)	0.77 (0.52-1.15)		
		No	311 (96.9)	96 (93.2)	407 (96.0)	Reference		
**Partner characteristics**								
	Age (years), mean (SD)		36.7 (5.5)	37.7 (6.2)	37.0 (5.6)		1.27 (306,96)	.12
	**Level of education, n (%)**							
		Primary	25 (7.9)	8 (7.9)	33 (7.9)	Reference		
		Secondary	134 (42.5)	47 (46.5)	181 (43.5)	0.98 (0.79-1.21)		
		Postsecondary	156 (49.5)	46 (45.5)	202 (48.6)	1.02 (0.83-1.25)		
		Missing	6	2	8			
	**Occupational status, n (%)**							
		Working	312 (98.7)	101 (100.0)	413 (99.0)	Reference		
		Not working	4 (1.3)	0	4 (1.0)	1.32 (1.25-1.40)		
		Missing	5	2	7			

The position most frequently used by women for delivering at home was on all fours; for delivering in a freestanding midwifery unit, it was squatting (Table 2). Delivering with two or more midwives was slightly more frequent in a freestanding midwifery unit, as was the use of a uterotonic agent (mainly oxytocin) at birth. None of the other monitored obstetric and neonatal parameters differed between the two delivery settings. No third- or fourth-degree perineal tears were observed in the studied population, and only two episiotomies were performed. Within one week of delivery, one mother and eight newborns were hospitalized, all after delivering at home, and all were discharged from the hospital after a few days.

**Table 2:** Birth-related characteristics and birth outcomes of women having planned births at home or in a freestanding midwifery unit.

Characteristics			Delivery location				Relative risk (95% CI)		*Z*		*F* (*df*1,*df*2)		*P* value
			At home (n=321)	In a freestanding midwifery unit (n=103)	Overall (N=424)								
**Birth-related**													
	Gestational age (weeks), median (IQR^a^)		40 (1)	40 (1)	40 (1)			0.75				.45	
	Birthweight (g), mean (SD)		3419.6 (451.4)	3397.1 (392.8)	3414.1 (437.3)					1.32 (312,102)		.22	
	**Small for gestational age, n (%)**												
		Yes	23 (7.2)	14 (13.6)	37 (8.7)	0.81 (0.62-1.04)							
		No	298 (92.8)	89 (86.4)	387 (91.3)	Reference							
	**Position in delivering, n (%)**												
		Lying down	46 (14.3)	30 (29.1)	76 (17.9)	0.94 (0.75-1.17)							
		Squatting	75 (23.4)	41 (39.8)	116 (27.4)	Reference							
		Kneeling	18 (5.6)	1 (1.0)	19 (4.5)	1.47 (1.23-1.74)							
		On all fours	130 (40.5)	16 (15.5)	146 (34.4)	1.38 (1.19-1.59)							
		On the side	32 (10.0)	15 (14.6)	47 (11.1)	1.05 (0.83-1.34)							
		Others	20 (6.2)	0	20 (4.7)	—^b^						—	
	**Number of midwives at delivery, n (%)**												
		1	23 (7.2)	1 (1.0)	24 (5.7)	1.29 (1.16-1.42)							
		≥2	298 (92.8)	102 (99.0)	400 (94.3)	Reference							
	Cord clamping (min), mean (SD)		84.2 (106.7)	88.8 (44.8)	85.3 (95.4)					1.25 (224,71)		.18	
	**Uterotonic agent use, n (%)**												
		Yes	98 (30.5)	45 (43.7)	143 (24.3)	1.43 (1.09-1.88)							
		No	223 (69.5)	58 (56.3)	281 (75.7)	Reference							
	**Lotus, n (%)**												
		Yes	76 (23.7)	27 (26.2)	103 (24.3)	0.97 (0.85-1.10)							
		No	245 (76.3)	76 (73.8)	321 (75.7)	Reference							
	**Exclusive breastfeeding at 10 days, n (%)**												
		Yes	314 (93.7)	100 (97.1)	414 (94.6)	Reference							
		No	7 (2.2)	3 (2.9)	10 (2.4)	0.92 (0.61-1.39)							
**Birth outcomes**													
	**Postpartum hemorrhage, n (%)**												
		≤500 mL	292 (91.0)	97 (94.2)	389 (91.7)	Reference							
		>500 mL	29 (9.0)	6 (5.8)	35 (8.3)	1.61 (0.65-3.98)							
	**Perineal tear (degree), n (%)**												
		None	171 (53.3)	57 (55.3)	228 (53.8)	Reference							
		First	103 (32.1)	33 (32.0)	136 (32.1)	1.01 (0.89-1.74)							
		Second	45 (14.0)	12 (11.7)	57 (13.4)	1.05 (0.90-1.23)							
		Third	0	0	0	—						—	
		Fourth	0	0	0	—						—	
	Mother’s postpartum hospitalization (within 1 week of delivery), n		1	0	1	—						—	
	Newborn’s hospitalization (within 1 week of birth), n		8	0	8	—						—	

^a^IQR: interquartile range.

^b^Cannot be calculated because the frequency is 0.

## Discussion

### Principal Results

The population enrolled in this first Italian study corresponded to 47% of the expected national deliveries over the study period and for all causes of out-of-hospital births. Considering that the investigated population was selected and only low-risk, planned out-of-hospital births were the target population, the findings are representative and support the choice of out-of-hospital planned births in Italy.

Being older than 35 years and being a primipara increased the probability of the delivery occurring in a freestanding midwifery unit compared to at home. Findings are in line with previous studies performed in different European countries, as well as in the United States and Canada [13-18]. Among the reasons that influence women in planning the place of birth are cultural attitudes, religion, and peer and family views, but another determinant guiding the choice to deliver out-of-hospital is also previous birth experience [19-21]. These reasons are valid for comparable settings in countries with middle and high availability of resources. In developing countries, where out-of-hospital birth rates are high, factors such as poverty, access to hospitals, and lack of transportation determine the choice [22,23].

### Strengths and Limitations

This study had limited power to detect small differences in variables with low incidence. As in similar countries [24], the rate of out-of-hospital births in Italy is low, so it is difficult to obtain large study groups. Therefore, the results of this study can be indicative for settings with similar services and societal structures. The strength of the study was its ability to obtain for the first time, using a rigorous data collection process, detailed information on out-of-hospital births in Italy for a large population that was based on a formal, updated, and evidence-based assistance protocol. The first national dataset was created and provided reasonable detail in terms of women’s characteristics, pregnancy monitoring, labor, birth, and neonatal outcomes of interest. These data have allowed the body of knowledge to be expanded by providing evidence on the results of comparisons performed between delivery settings.

### Conclusions

This study made it possible, for the first time, to obtain a large amount of information about out-of-hospital births in Italy. The tools used for data collection and visualization have allowed us to optimize the acquisition and monitor the information. The future goal is to be able to expand this data collection to all out-of-hospital births in the national territory. This would also allow midwives to have a more complete and detailed view of the work they perform and help monitor and improve the clinical practice of out-of-hospital births. Moreover, with an increase in the number of enrolled midwives, it would be possible to use the portal not only as a Web-based form but also as a portal for sharing resources that would help midwives in their clinical practice. The interactive graphs and maps [25] used for visualizing and processing data can be valuable instruments for sharing results.

## Abbreviations

CeDAP: birth assistance certificate [Certificato Di Assistenza al Parto]
CRF: case report form
IQR: interquartile range
RR: relative risk
